# An Overview Regarding Microbial Aspects of Production and Applications of Bacterial Cellulose

**DOI:** 10.3390/ma15020676

**Published:** 2022-01-17

**Authors:** Raluca Elisabeta Lupașcu, Mihaela Violeta Ghica, Cristina-Elena Dinu-Pîrvu, Lăcrămioara Popa, Bruno Ștefan Velescu, Andreea Letiția Arsene

**Affiliations:** 1 Department of General and Pharmaceutical Microbiology, Faculty of Pharmacy, “Carol Davila” University of Medicine and Pharmacy, 6 Traian Vuia Street, 020956 Bucharest, Romania; andreea.arsene@umfcd.ro; 2 Department of Physical and Colloidal Chemistry, Faculty of Pharmacy, “Carol Davila” University of Medicine and Pharmacy, 6 Traian Vuia Street, 020956 Bucharest, Romania; mihaela.ghica@umfcd.ro (M.V.G.); cristina.dinu@umfcd.ro (C.-E.D.-P.); lacramioara.popa@umfcd.ro (L.P.); 3 Department of Pharmacology and Clinical Pharmacy, Faculty of Pharmacy, “Carol Davila” University of Medicine and Pharmacy, 6 Traian Vuia Street, 020956 Bucharest, Romania; bruno.velescu@umfcd.ro

**Keywords:** cellulose, bacterial cellulose, biomedical applications, wound dressing

## Abstract

Cellulose is the most widely used biopolymer, accounting for about 1.5 trillion tons of annual production on Earth. Bacterial cellulose (BC) is a form produced by different species of bacteria, representing a purified form of cellulose. The structure of bacterial cellulose consists of glucose monomers that give it excellent properties for different medical applications (unique nanostructure, high water holding capacity, high degree of polymerization, high mechanical strength, and high crystallinity). These properties differ depending on the cellulose-producing bacteria. The most discussed topic is related to the use of bacterial cellulose as a versatile biopolymer for wound dressing applications. The aim of this review is to present the microbial aspects of BC production and potential applications in development of value-added products, especially for biomedical applications.

## 1. Introduction

Plants are the major contributor of cellulose and the first report on the discovery of cellulose produced by bacteria, especially by *Acetobacter xylinum*, was made by A.J. Brown in 1986. In 1988, he observed the development of an unbranched film with chemically equivalent structure as plant cellulose. Although Brown talked about bacterial cellulose (BC) since 1986, major studies regarding the subject began in the 21st century [1].

Recent studies show that bacterial cellulose can be produced by both Gram-positive and Gram-negative bacteria. Bacterial sources for production of BC with different structures and biological roles are: *Aerobacter*, *Alcaligenes*, *Pseudomonas*, and *Achromobacter* with the biological role of flocculation in wastewater; *Agrobacterium* and *Rhizobium* with the biological role of attachment to plants; and *Gluconacetobacter* with the biological role in aerobic environments [2]. The structure of bacterial cellulose consists of glucose monomers that give it excellent properties for different medical applications (unique nanostructure, high water holding capacity, high degree of polymerization, high mechanical strength, and high crystallinity). These properties differ depending on the cellulose-producing bacteria [2].

In recent years, there have been many reviews about the production of bacterial cellulose and the various areas of application in the medical field. The most discussed topic is related to the use of bacterial cellulose as a versatile biopolymer for wound dressing application. The aim of this review is to present the microbial aspects of BC production and potential applications in development of value-added products.

## 2. Bacterial Cellulose—Structure and Characteristics

Bacterial cellulose has a chemical structure similar to the structure of plant-derived cellulose, but is devoid of lignin, pectin, and hemicellulose—components that are associated with plant cell wall structure [3]. At the same time, the structural differences between BC and cellulose derived from plants are observed due to the structural arrangement of BC fibers which gives them mechanical properties, the absence of contaminating polymers and a higher crystallinity [4].

The basic structure of bacterial cellulose consists of β-1 → 4 glucan chains that are held together by inter- and intra-hydrogen bonds, having the following molecular formula: (C_6_H_10_O_5_)_n_.

**Figure 1:**
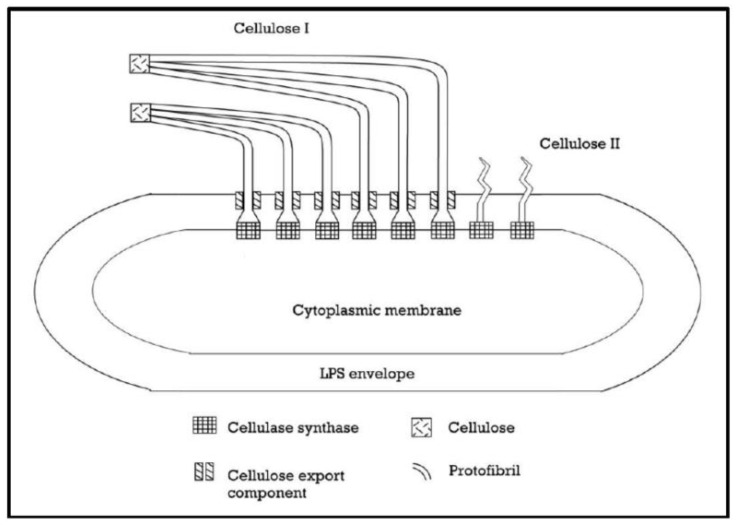
Production of cellulose microfibrils by *Acetobacter xylinum* [1].

*Acetobacter xylinum* produces cellulose I (ribbon-like polymer) and cellulose II (thermodynamically stable polymer) as described in Figure 1. During the synthesis process, protofibrils of glucose chain are secreted through bacteria cell wall and aggregate together forming nanofibrils cellulose ribbons which form the web shaped network structure. The cellulose formed has an abundant surface of hydroxyl groups that explaining its hydrophilicity, biodegradability, and chemical-modifying capacity [1].

**Figure 2:**
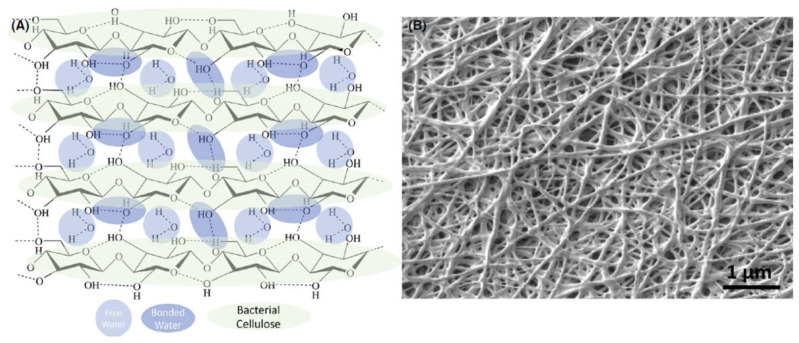
Bacterial cellulose (BC). (**A**) Molecular structure of hydrated BC. (**B**) Typical microscopic BC fiber film morphology [4].

The water retention capacity (WHC) and water release rate (WRR) of the BC are dependent on the pore volume and the available surface area. The lower the pore and surface area of the BC structure, the lower the WHC values and the lower the WRR values. The denser microfibrils result in a greater amount of water retained in the system due to the formation of hydrogen bonds and a smaller amount of free bulk water, which prevents water evaporation (Figure 2).

In recent years, there have been many studies on the link between the structure of BC and the ability to retain water [4]. These studies show that if the size of the pores and surface area of BC is decreased, WHC decreases and WRR increases. To reduce the dimension of pores and surface it can be used single sugar-linked glucuronic acid-based oligosaccharide (SSGO) in the culture media. BC porosity can also be reduced in the presence of hydroxypropyl methylcellulose (HPMC), resulting in a low WHC value. If carboxymethylcellulose (CMC) is used in the culture media, a fibrillary network with larger ribbons is formed due to the adhesion of CMC on the BC surface, thus resulting in higher WHC.

Due to its intrinsic properties (hydrophilicity, porosity, and biocompatibility) and web shaped network structure, bacterial cellulose is of interest for use as wound dressing material since it allows the controlled release of substances [4].

## 3. Bacterial Cellulose—Production

The process of synthesis of BC is complex and specific, comprising several stages. In the first stage, the synthesis of uridine diphosphoglucose (UDPGlc) is performed, then the polymerization of glucose from the β-1 → 4 glucan chain, which is a linear chain [5]. In the process of synthesis of uridine diphosphoglucose (UDPGlc) there are three stages: phosphorylation of glucose by glucokinase, isomerization of glucose-6-phosphate into glucose-1-phosphate by phosphoglucomutase, and synthesis of UDP-glucose by uridylyltransferase (UTP)-glucose-1-phosphate.

The free hydroxyl groups in the lateral and unidirectional chains form inter- and intra-chain hydrogen bonds, the chains turning into insoluble nanofibrils up to 1–9 m long, representing 2000–18,000 glucose residues. Properties of BC, as high-water retention capacity, hydrophilicity, and crystallinity, are the result of the formed nanofibrils that cluster in ribbon-shaped fibrils.

The most known bacterium producing BC is *Komagataeibacter xylinus* (former name *Acetobacter xylinum*) which belongs to the group of acetic acid bacteria (AAB) which are Gram-negative bacteria and strictly aerobic. The reaction conditions by which this bacterium ferments are pH 3–7 and the temperature between 25–30 ℃, using saccharides as a carbon source [6].

**Figure 3:**
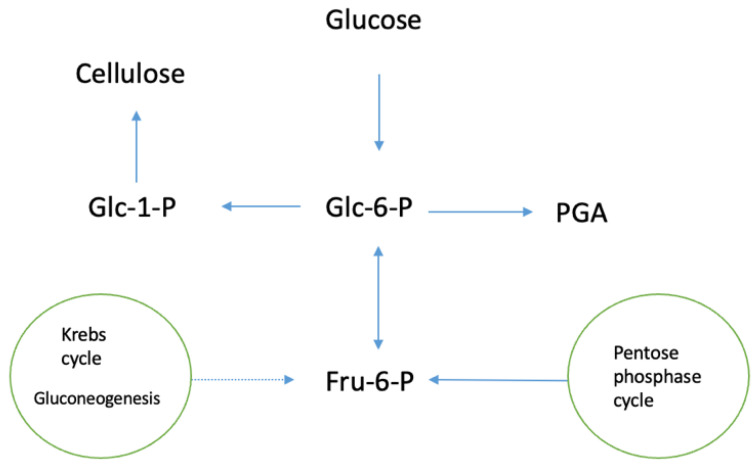
Pathways of carbon metabolism in *Acetobacter xylinum*, CS, cellulose synthase (adapted from S. Bielecki, A. Krystynowicz, M. Turkiewicz, H. Kalinowska, Bacterial Celluose) [5].

*Acetobacter xylinum* has an efficiency of about 50% for the transformation of various carbon compounds into cellulose, the final product of carbon metabolism. This process involves the pentose phosphate cycle, or the Krebs cycle coupled with gluconeogenesis, as it is summarized in Figure 3.

Fermentation of BC production can be achieved in static, agitated, or stirred conditions, resulting different forms of cellulose. If the BC is produced in static conditions, the yield is dependent on the concentration of the carbon source and will result a three-dimensional interconnected reticular pellicle. The process is regulated by air supply from medium surface, with bacteria becoming inactive due to insufficient oxygen supply. The process under static conditions it is recommended for production of BC at an industrial scale.

Both agitated and stirred conditions produce irregular shaped sphere-like cellulose particles (SCP), such as fibrous suspension, spheres, pellets, or irregular masses. Even if the entire process of SCP formation still remains unknown and the SPC has lower crystallinity, mechanical strength, and degree of polymerization, most cellulose used in commercial applications is generated through agitated fermentation [1].

BC production is an expensive process, primarily due to the culture medium, which represents approximately 30% of the total cost of production and due to the low productivity of known bacterial strains. In view of the fact that there is interest in identifying an efficient and cost-effective culture medium, many researchers have tried to obtain BC from alternative culture media [1]. The best sources of carbon for BC production are glucose, fructose, and glycerol, but to reduce production costs, most researchers recommend the use of various waste products such as fruit juices, spruce hydrolysate, wine fermentation waste broth, rotten fruit, cotton-based waste textiles, and wheat straw [7]. In recent years, there have been many experimental studies conducted on the production of bacterial cellulose.

Costa et al. (2018) tried to demonstrate how BC can be obtained from *Gluconacetobacter hansenii* UCP1619 using industrial residues as nutrients. They used Hestrin–Schramm (HS) culture medium and other medium formulated with different sources of carbon (sugarcane molasses and acetylated glucose) and nitrogen (yeast extract, peptone and steep corn liquor (CSL)). Among the alternative culture media, only the one formulated with 1.5% glucose and 2.5% CSL functioned as a substitute for carbon and nutrients, the mass of BC obtained corresponding to 73% of the mass obtained on HS medium. The BC films obtained had a homogeneous, compact, three-dimensional structure, those obtained on the alternative culture medium had a higher thermal stability than those obtained on the HS medium. The dry and hydrated BC films were also put through to the mechanical tensile test, the dry film having a rigidity 96.98% higher than the hydrated film [7,8].

Another study conducted on other strain of *Gluconacetobacter* was made by Revin et al. in 2018. The aim of the study was to investigate the production of BC by *Gluconacetobacter sucrofermentans* strain B-11267 using thin stillage (TS) and whey, without pretreatment or addition of other nutrient sources. TS is obtained as a byproduct of microbial ethanol fermentation of carbohydrates and subsequent distillation of the fermented mash. The HS medium was used as the comparison medium.

The bacterial cultivation of *Gluconoacetobacter sucrofermentans* B-11267 was obtained as follows: 1 mL suspension of Kombucha tea was added to a test tube with 9 mL of 0.9% sodium chloride (*w*/*v*). Several dilutions were performed and an aliquot of 0.1 mL of each dilution was spread on the culture medium made from glucose (100 g/L), yeast extract (10 g/L), calcium carbonate (20 g/L), and agar (15 g/L) and the plates were incubated at 28 °C for 72 h. The colonies with a clear zone around were selected and transferred to tube containing 10 mL of Hestrin and Schramm (HS) medium.

After the incubation period it was observed that the highest yield of BC (6.19 g/L) was obtained using TS on the culture medium—with whey, 5.45 g/L was obtained; and on the HS medium, 2.14 g/L was obtained.

**Figure 4:**
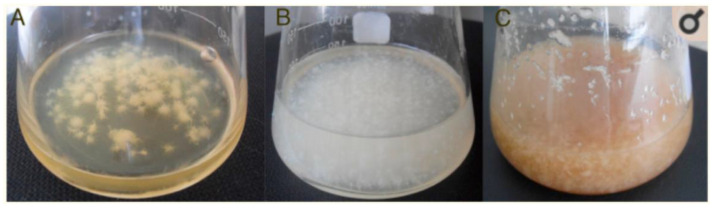
BC produce by *Gluconoacetobacter sucrofermentas* B-11267 in agitated culture conditions using HS medium (**A**), whey (**B**), and thin stillage (**C**) [6].

It is known that the composition of the nutrient medium impacts the structure of cellulose [8]. This study shows that the cellulose obtained on the TS and whey medium was more homogenous and finer than the ones from HS medium, as it can be seen on Figure 4 [8].

Another cost-effective method for production of BC is polysaccharide fermentation waste which are rich in extracellular polysaccharides, low-molecular weight sugars, glycerol, proteins, vitamins, and other nutrients. Zhao et al. (2018) had demonstrated in their study that the use of pullulan polysaccharide fermentation wastewater presented great results as a low-cost substrate for BC production. The polysaccharide fermentation waste presents high chemical oxygen demand (COD) values, which, during the fermentation, decreased, meaning that this source of nutrients could be an efficient economic approach for obtaining high value-added materials with minimum pollution [9].

In addition to those presented above, there are many other studies that highlight various nutrient sources that can be used for BC production. In 2013, Raghunathan published a paper regarding the production of microbial cellulose from the new bacterial strain isolated from temple wash waters. Another study by Costa et al. (2018) presents the benefits of using corn steep liquor as nutrient sources for production of BC. The use of different waste products from agricultural and industrial activities could improve the sustainability of BC production and reducing environmental pollution [7,10].

## 4. Biomedical Applications of Bacterial Cellulose

From an application standpoint, BC has features that have application potential in many industries—such as the food industry, paper, composition and cosmetics, and biomedical applications. Given the areas of applicability, many researchers have focused on the development of BC-based products; in the recent literature, many biomedical applications have examined and documented [3].

Bacterial cellulose has a structure very similar to collagen, being used in tissue engineering, cells—such as artificial skin, vascular grafts, artificial bones, and cartilage, etc. BC is used quite often to treat wounds because it helps maintain a moist environment with the ability to retain residues. In patients who have had wounds treated for a long time with BC-based wound dressings, the activity of proteolytic enzymes, cytokines, and reactive oxygen species has been reported [6].

### 4.1. Dermal Applications

The skin plays an important physiological role in a person’s everyday life. This physiological role is essentially to protect the organism. The skin is a structured and continuously adaptable barrier between the organism’s internal environment and its surroundings. Pathological changes in the skin will logically be the result of external or internal aggressions. The thickness of the skin varies considerably based on body area, gender, and age. The skin has a relatively complex structure. It consists of four main layers: the epidermis (outer layer), the dermo-epidermal junction with the basal membrane, the dermis, and the hypodermis.

It is very important to develop materials that can expedite the wound healing process, since a very big disadvantage that comes with the long-wound healing time is the adhesion and growth of bacterial cultures on the wound surface. This leads to the proliferation of bacteria that is not beneficial to the patient’s health, especially as in recent years the antibiotic resistance has greatly increased. The development of dressing materials with antimicrobial substances or other bioactive properties significantly reduced the complications caused by bacterial infection of wounds [11].

The most common skin lesions are burns, cuts, and scratches that can cause extensive destruction of skin tissue. The wound healing process comprises several stages: hemostasis, inflammation, granulation tissue growth (proliferation), and remodeling (maturation). Winter (1962) was the first to notice that re-epithelialization and wound healing was much faster if a humid environment is maintained [12]. There are many studies showing that the utilization of BC-based wound dressing materials has accelerated the healing of wounds caused by second-degree burns. Czaja et al. (2007) stated that the patients in their study were divided into two groups, one group used the BC membrane to treat burns, and the other group used conventional dressings. They have found that non-dried BCs maintained a suitable water balance that considerably reduced wound pain and increased wound healing [13].

These beneficial effects of BC-based materials were also demonstrated by Fu et al. (2012) in an animal study. They reported that BC-based dressings reduced the inflammatory process and accelerated the regeneration and healing of wounds [14]. In another study, Fu et al. used BC as a replacement for gauze bandage to heal skin tissues in vivo. From a pathological point of view, differences were identified in the wound healing process depending on the application of BC. BC applied in a thicker layer has shown faster effects of reducing inflammation and accelerating healing than BC applied in a thinner layer [15].

The first BC-based dressing material to appear on the market was Biofill^®^. It is used as a temporary substitute for the skin, being a very thin film containing 8.5% water, or as a biological dressing. The only disadvantage of this product is the low elasticity, which was observed when the dressing was applied in areas with high mobility. The advantages of the Biofill^®^ product include immediate pain relief, easy removal of the dressing, fast healing, tight adhesion to the surface of the lesion, and low cost [16,17].

**Figure 5:**
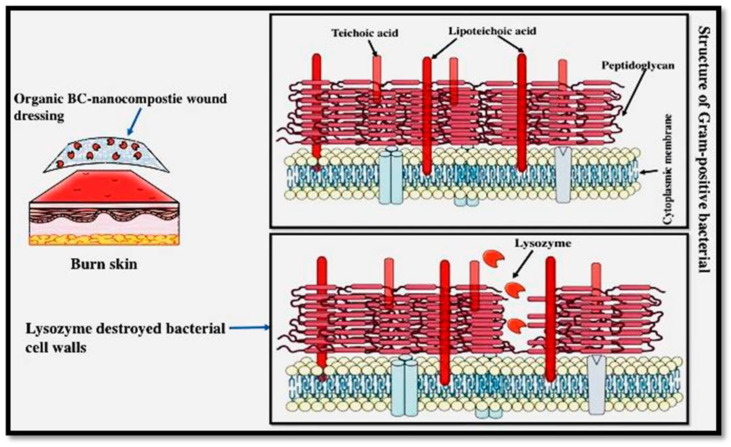
Organic nanocomposites for wound healing [11].

Given the above, regarding the development of materials with antimicrobial substances, BC-based materials do not inhibit wound infection. To provide protection against bacterial proliferation, BC-based materials are produced in combination with antimicrobial agents, by physical or chemical methods. Depending on the antimicrobial agents used, BC-based materials are classified into two categories: BC incorporated with inorganic materials (i.e., Ag, ZnO, and CuO particles, and their derivatives) and BC incorporated with organic antimicrobial agents (e.g., lysozyme). In Figure 5, it can be seen that the modified BC is prone to lysozyme decomposition [11].

Since ancient times, silver and its compounds have been shown to be effective in preventing bacterial infections due to its antibacterial, antifungal, and antiviral properties [18]. Thus, there have been many attempts to incorporate silver ions into BC-based materials and it has been shown that the effectiveness of Ag nanoparticle in BC-based materials is influenced by the size and shape of the nanoparticles [19,20].

Silver ions act through two mechanisms of action to destroy bacteria: by interacting with enzyme and protein thiol groups, which are significant elements for bacterial respiration and transport of the essential substance through the cell wall and inside the cell; modifying the function of the cell wall by attaching silver ions to the bacterial cell membrane and to the outer bacterial cell.

Increasing the effectiveness of BC dressing materials can be achieved by modifying cellulose in the manufacturing process, without changing the biocompatibility and nanofibrillar structure. Several recent findings regarding several modifications of BC can be seen in Table 1.

### 4.2. Ophthalmology

Amniotic membrane dressings are used to heal ocular surface, and if the reconstruction of the eye surface is necessary, a corneal transplant is performed. Ophthalmology is a branch of medicine for which innovative approaches to regenerative medicine are beginning to reveal interest. In ophthalmology, biologically derived materials are used for the ocular surface, less often for the retina. Due to its hydrophilicity, flexibility, and mechanical stability, BC is a candidate for corneal regeneration [35,36].

In recent years, collagen-based materials have been developed for reconstructing the cornea or conjunctival epithelium or even materials based on silk and keratin. However, BC by its conformability increases the adaptability of the biomaterial to the dome shape of the ocular surface. To increase the long-term efficacy of BC applied in corneal regeneration in the fabrication process, BC was combined with polyvinyl alcohol (PVA) by a freeze–thaw method [21,37]. BC/PVA-based materials have the following important characteristics for corneal replacement: mechanical properties and thermal stability, light transmission, and water retention capacity. Research on the applicability of BC in ophthalmology is rare, although many preliminary results show satisfactory results [37,38].

### 4.3. Tissue Engineering

Tissue engineering is a field for which BC, due to its properties, is of significant interest, especially as an alternative to cartilage. In tissue engineering, materials are used in a way that allows the interaction between scaffold materials, the cells, and the surroundings in order to be able to develop a new tissue. Due to the porous structure, including the structure of the 3D network, biocompatibility, and low cytotoxicity, many researchers have stated that BC-based materials are best suited for use in tissue engineering [39,40].

A study was conducted to investigate PVA-BC nanocomposites as potential substitute materials for articular cartilage. In this study, BC was obtained from *Gluconoacetobacter xylinus* before being crushed into powder. PVA, sodium alginate, and carboxy methyl cellulose were incorporated into the BC suspension. The mixture was then poured and freeze-dried to obtain the artificial endocranium (AE). PVA-BC nanocomposites showed elasticity close to that of the initial cartilage, having potential for several orthopedic problems, including the replacement of damaged intervertebral discs. Due to its high mechanical properties, low cost, and elasticity, AE has been prepared and then patented [41,42].

### 4.4. Blood Vessel Replacement

The blood vessels are the components of the circulatory system that transport blood throughout the human body. These vessels transport blood cells, nutrients, and oxygen. They also take waste and carbon dioxide away from the tissues. In certain pathological conditions, the blood vessels can become clogged or damaged, which makes it difficult for the body to function normally. In these situations, the blood vessels are replaced with other blood vessels from the patient or donor or, due to the evolution of medicine, artificial blood vessels are used.

**Figure 6:**
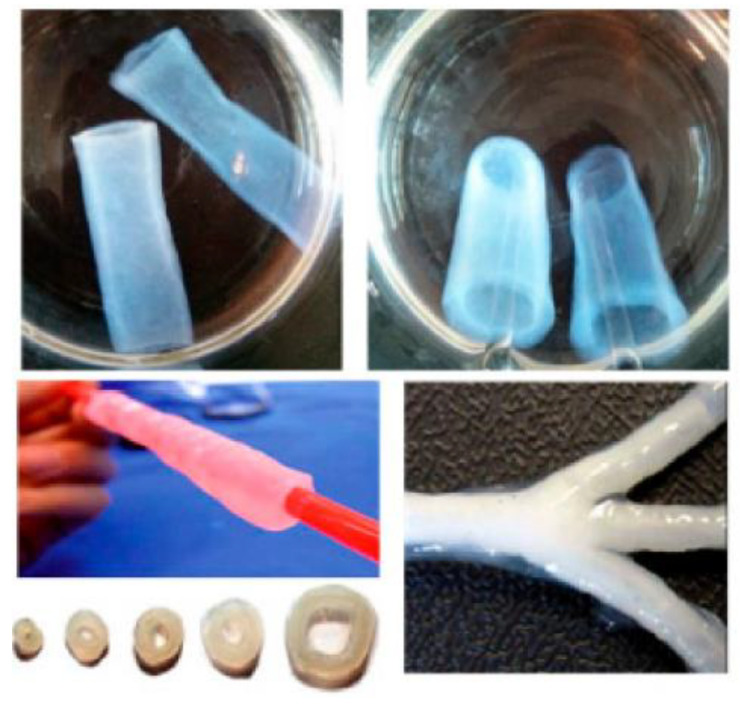
Vascular graft and blood vessel tubes with different sizes and shape, produced by fermentation onto a branched silicone tube [6].

Unfortunately, the existing artificial blood vessels cannot be used in the case of small blood vessels with a diameter of 6 mm. BC has a breaking pressure of up to 800 mmHg which makes it ideal for the manufacture of artificial blood vessels (Figure 6) that can be used for both small and larger vascular grafts [40,43,44]. Pure BC or combined with graphene oxide (GO) can be used to produce artificial blood vessels, studies have shown that BC combined with GO presented a higher biocompatibility than applied individually. In another study, paraffin wax was used in a culture medium with *Gluconoacetobacter xylinus* to obtain 3D network scaffolds with adjustable microporosity. These microporous scaffolds can be used for the proliferation, differentiation, and migration of endothelial cells, being a candidate for the constitution of artificial blood vessels [45,46].

According to the World Health Organization (WHO), cardiovascular disease (CVD) is the leading cause of death globally, with 17.9 million deaths annually. The most common heart surgery is coronary bypass graft surgery, which is performed to improve blood flow to the heart muscle. The procedure involves taking a healthy blood vessel from leg, arm, or chest and connecting it beyond the blocked arteries in your heart.

There are several artificial grafts obtained from polytetrafluoroethylene, polyethylene, polyethylene terephthalate, and polyurethane, but which are not suitable for vascular surgery, which requires harvesting vessels from the thorax or leg. Researchers at Dieter Klemm (2010) were the first to attempt the use of vascular substitutes obtained from BC, obtaining the product bacterial synthesized cellulose (BASYC) which has been successfully applied as a synthetic blood vessel in animal models for microsurgery. BASYC has a high mechanical resistance in the wet state and high-water conservation. They checked if there are some coagulation problems induced by the biomaterial with BC, the biological properties of the tubes, cell adhesion, and proliferation, ultimately finding that BC have great potential for tissue engineering [47,48].

### 4.5. Drug Delivery

Another application of BC-based materials is drug delivery systems. Drugs that require administeration in controlled doses are difficult to formulate and the costs are high, which is why bacterial cellulose has been widely studied to facilitate the controlled administration of drugs. Many studies refer to manufactured BC compositions used as a carrier for the administration of transdermal drugs [49,50].

To evaluate the therapeutic efficacy of BC membranes, an in vitro penetration study was performed for two different substances: lidocaine hydrochloride and ibuprofen. The diffusion of the two drugs was investigated by the diffusion cell technique. The release of the two substances from the BC compounds were analyzed in comparison with other conventional formulations: ibuprofen gel, lidocaine hydrochloride gel, and solution in PEG400. It was found that the use of lidocaine hydrochloride in BC compounds resulted in a lower penetration rate compared to conventional preparations. The results are also due to the increased porosity of BC-based materials [23,49,50].

Another group of researchers reported high antibacterial activity against *Escherichia coli*, *Pseudomonas aeruginosa*, *Staphylococcus aureus*, and *Citrobacter freundii* when using BC nanocomposites incorporated with zinc oxide nanoparticles. BC/ZnO nanocomposites were applied to the surface of a burn wound on animal models. After 15 days, nanocomposites with BC/ZnO showed a cure rate of 66% compared to BC, which showed a cure rate of 50.5% [24].

Khalid et al. (2017) showed good results related to the incorporation of TiO_2_ in BC-based materials. They also did an in vivo study on the human epidermis to see the rate of wound healing after burns. The antimicrobial activity of the nanocomposite was much higher in the case of BC/TiO_2_ compared to BC [25]. In their study, Mueller et al. (2013) demonstrated the efficacy of using BC-based materials for the administration of serum albumin proteins. It was found that the freeze-dried BC materials had exhibited a lower loading of protein compared to the normal BC ones [51]. Other groups of researchers have tested the integration of drug molecules into the BC material to allow a gradual release of the drug [52].

A remarkable compatibility with BC-based materials is presented by active pharmaceutical ingredients (APIs) derived from plants. There is a study which demonstrates the improvement of the antibacterial and antioxidant activity of silymarin-zein nanoparticles if they are incorporated with BC nanofibers [44,53].

In the current state, systems with long-term controlled release of active pharmaceutical ingredients (APIs) are very rare, which is why Alkhatib et al. (2017) have developed a release system consisting of BC and Poloxamer for the treatment of wounds with octenidine. The system developed by them offers a retention time of up to a week, improving the antimicrobial and antiseptic properties of octenidine [25,27].

All of these studies demonstrate the remarkable properties of BC for the development of drug delivery systems, which offer many advantages: increased patient compliance, therapeutic efficacy, and relative stability of plasma concentration. Mostly, the water content of the BC membranes was partially lower due to absorption of a solution of the particular drug with glycerol, which causes a plasticizing effect. Regarding this, it can be concluded that BC membrane is more flexible and appropriate for transdermal applications [36,37].

## 5. Future Advancements

The applicability of bacterial cellulose as a wound management system became at-tractive for pharmaceuticals and biotechnology companies. It is estimated that by 2023, in America, the total market value of microbial cellulose will be $570 million, which rep-resents an increase of 14.8% [53]. Medicine is evolving substantially every day and one of the most beneficial evolutions it is represented by personalized medicine. Every illness acts differently from one person to another and individual treatment regimen with specific factors are required. For a deeper understanding of the importance to embrace personalized medicine, we can think of diabetes, where every patient presents different forms of wounds or ulcers and needs to be treated following the patients’ parameters. Due to its properties, bacterial cellulose could respond to the necessity of developing individual bacterial cellulose wound dressings that are capable of releasing dose-specific medications directly to the wound site, all whilst monitoring the healing process of the wound in real-time, without the need to change the dressing [26,54].

Even if bacterial cellulose has a wide-reaching gamut of applications in biotechnological and biomedical industry, not all results were aligned with the predictions of researchers, which raised some doubts. For biomedical applications, issues are in the area of storage, handling, and production of bacterial cellulose derivatives, because the technique of bioprinting seems that considerably lowers the tensile strength of the finished printed product [53]. Another future advancement for bacterial cellulose is represented by the necessity of alternative methods of sterilization, such as radiation. Bacterial cellulose is highly stable under high heat, but its bioactive components are sensitive at high temperatures and become inactive, rendering the material ineffective for its targeted use. In order to use BC materials as tissue grafts and wound dressings, it is crucial to develop a satisfactory sterilization method [54].

Furthermore, microbial cellulose films—having an interesting nanostructure—seem to have numerous other applications in wound recuperating and regenerative medicine, such as guided tissue recovery (GTR), periodontal medications, or as a substitution for dura mater (a film that encompasses brain tissue) [13].

## 6. Conclusions

Due to its very thin reticular structure, BC has unique physico-chemical properties—such as purity, high porosity, permeability to liquids and gases, fast water absorption capacity—which make it much more advantageous than vegetable cellulose. The structural differences between BC and cellulose derived from plants are observed due to the structural arrangement of BC fibers which gives them mechanical properties, the absence of contaminating polymers, and a higher crystallinity.

For a wider application of BC, the production process has been precisely studied by researchers, because BC production is an expensive process due to the culture medium. In this overview, we presented some of the attempts of researchers to obtain alternative culture media, which would lead to a more cost-effective production. The results of these studies show that the waste products that can be used for BC production are: industrial residues, polysaccharide fermentation waste, corn steep liquor, thin stillage, and whey. The use of different waste products from agricultural and industrial activities could improve the sustainability of BC production and reduce environmental pollution.

Another topic that we presented in this overview regards the possibility of applying BC in the medical field. We presented the BC applications regarding dermal applications, ophthalmology, tissue engineering, blood vessel replacement, and drug delivery systems. The most discussed application of BC refers to wound healing, BC-based wound dressing materials accelerate the healing of wounds caused by second-degree burns, by reducing the inflammatory process and accelerating the regeneration and healing of wounds. Satisfactory results were presented for ophthalmology, where BC is a candidate for corneal regeneration. The biocompatibility and mechanical properties of BC make it an ideal material for pursuing further biomedical research and applications.

Bacteria produce a large number of compounds with high diversity in biology, medicine, and the pharmaceutical industry. Some bacteria produce bacteriocins, others produce compounds with vitamin rolls or produce inosine which is present in some dietary supplements [55], but the discovery of cellulose produced by bacteria had definitely changed these industries.

## Figures and Tables

**Table 1:** Modifications of bacterial cellulose (BC) and properties resulting from the modifications [15].

Material	Title of Paper	Results Obtained by BC Modification	References
BC with structured topography	Surface-structured bacterial cellulose with guided assembly-based biolithography (GAB)	Improved cell alignment. Promotion of fibroblast infiltration and new collagen deposition in the wound bed.	[21]
BCNC/RC	Regenerated chitin fibers reinforced with bacterial cellulose nanocrystals as suture biomaterials	Biocompatible surgical sutures Increasing strength of BCNC/RC filaments. Enzymatic degradation possible. Degradation rate can be tuned by varying concentration of BCNCs in the yarn. Chitin can promote cell proliferation (in vivo).	[22]
TOBCP/AgNP	TEMPO-oxidized bacterial cellulose pellicle with silver nanoparticles for wound dressing	Antimicrobial activity Ag^+^ release with a rate of 12.2%/day at 37 °C in 3 days biocompatible wound dressing.	[23]
BC/ZnO	Bacterial cellulose-zinc oxide nanocomposites as a novel dressing system for burn wounds	Antimicrobial activity against *Escherichia coli*, *Pseudomonas aeruginosa*, *Staphylococcus aureus*, and *Citrobacter freundii.* Significant healing of 66% after 15 days related to day 0.	[24]
BC/TiO_2_	Bacterial cellulose-TiO_2_ nanocomposites promote healing and tissue regeneration in burn mice model	Antimicrobial activity against *Escherichia coli* (81.0 ± 0.4%) and *Staphylococcus aureus* (83.0 ± 0%).	[25]
BC/SMN-Zein	Drug release and antioxidant/antibacterial activities of silymarin-zein nanoparticle/bacterial cellulose nanofiber composite films	Flavonoid silymarin (SMN) and zein loading through nanoparticle adsorbing onto BC nanofibers. Change of wettability and swelling. Antioxidant and antibacterial activity air-dried SMN-Zein/BC nanocomposite slow down the lipid oxidation.	[26]
BC/Octenidin	Controlled extended octenidine release from a bacterial nanocellulose/poloxamer hybrid system	Long term-controlled release of octenidine up to one-week improved mechanical and antimicrobial properties. Ready-to-use system with poloxamer loaded BC for advanced treatment of infected wounds. Toxicity test performed with shell-less hen’s egg model.	[27]
BC/CMC/MTX	Effect of in situ modification of bacterial cellulose with carboxy-methylcellulose on its nano/microstructure and methotrexate release properties	Impact of DS-CMC on methotrexate loading. Topical treatment of psoriasis. Decrease of the elastic modulus as the DS of CMC increased.	[28]
BC/PHEMA	Embedding of bacterial cellulose nanofibers within PHEMA hydrogel matrices: tunable stiffness composites with potential for biomedical applications	New modification: in situ UV radical polymerization of HEMA monomer impregnated into wet BC nanofibrous structure. Significant improvement in mechanical properties. Tensile strength increased. Nontoxic. rMSCs (rat mesenchymal stem cells) proliferation. Tissue replacement and wound healing.	[29]
BC/ε-poly-L-Lysine	Functionalization of bacterial cellulose wound dressings with the antimicrobial peptide ε-poly-L-Lysine	Antimicrobial activity (broad-spectrum) without affecting the beneficial structural and mechanical properties Modification with non-toxic biopolymer ε-PLL inhibited growth of *S. epidermidis* on the membranes but did not affect the cytocompatibility to cultured human fibroblast.	[30]
BC/PVA	Preparation and in vitro characterization of BC/PVA hydrogel composite for its potential use as artificial cornea biomaterial	Higher visible light transmittance than plain BC.	[31]
BC/HA	Bacterial cellulose/hyaluronic acid composite hydrogels with improved viscoelastic properties and good thermodynamic stability	Higher visible light transmittance than plain BC.	[32]
ABC/urinary bladder matrix	Acetylated bacterial cellulose coated with urinary bladder matrix as a substrate for retinal pigment epithelium	Higher adhesion and proliferation of retinal pigment epithelium cells than uncoated BC. Closer recapitulation of the in vivo cell phenotype than uncoated BC.	[33]
BC/varying porosity	Bacterial cellulose-based biomimetic nanofibrous scaffold with muscle cells for hollow organ tissue engineering	Higher pore size than native BC to allow muscle cell ingrowth. Higher porosity. Small decrease in mechanical strength.	[34]
